# Publication status and reporting quality of case reports on acupuncture-related adverse events: A systematic reviews of case studies

**DOI:** 10.1016/j.heliyon.2023.e20577

**Published:** 2023-09-30

**Authors:** Tae-Hun Kim, Myeong Soo Lee, Stephen Birch, Terje Alræk, Arne Johan Norheim, Jung Won Kang

**Affiliations:** a Korean Medicine Clinical Trial Center, Korean Medicine Hospital, Kyung Hee University, Seoul, Republic of Korea; b Division of Clinical Medicine, Korea Institute of Oriental Medicine, Daejeon, Republic of Korea; c School of Health Sciences, Kristiania University College, Oslo, Norway; d Department of Community Medicine, Faculty of Medicine, National Research Center in Complementary and Alternative Medicine, UiT the Arctic University of Norway, Tromso, Norway; e Department of Acupuncture & Moxibustion, College of Korean Medicine, Kyung Hee University, Seoul, South Korea

**Keywords:** Acupuncture-related adverse events, Adverse events, Case report, Causality, CARE statement, Systematic review

## Abstract

**Introduction:**

Case reports on acupuncture-related adverse events (AEs) have been consistently published in the literature. This review aims to assess the current publication status of case reports on acupuncture-related AEs and evaluate their reporting quality in order to identify areas for improvement.

**Methods:**

This study is a systematic review (SR) of case reports. Studies describing cases for acupuncture-related AEs between 2010 and 2023 (until July) were searched in PubMed, Embase, and local databases (China and Korea), as well as by hand-searching references included in published relevant SRs. A bibliometric analysis was conducted to examine the publication trends of the included literature. The appropriateness of the acupuncture described in the cases, the causality assessment between AEs and acupuncture treatment, and the presence of necessary items from the CAse REport guidelines (CARE) checklist were narratively analyzed.

**Results:**

A total of 169 case reports were included in this review. Over the past decade, an average of 12 case reports on acupuncture-related AEs were published annually. However, only 38.2% of the articles provided sufficient information to determine the appropriateness of the acupuncture treatment used in the reported cases, and considerable numbers of the included case reports did not suggest enough information for the assessment of a causal relationship. The majority of cases did not report the timeline (n = 164), patient perspectives (n = 157), and informed consent (n = 121) items from the CARE checklist.

**Discussion:**

Acupuncture-related AEs persist in being frequently reported in the literature. Nonetheless, the information concerning acupuncture and causality assessment within these publications is still found to be insufficient. The development of reporting guidelines for future case reports on acupuncture-related AEs is anticipated to promote an academic environment conducive to more comprehensive reporting.

## Abbreviations

Adverse events: (AEs)
CAse REport guidelines: (CARE)
China National Knowledge Infrastructure: (CNKI)
Complementary and alternative medicine: (CAM)
Oriental Medicine Advanced Searching Integrated System: (OASIS)
Randomized controlled trials: (RCTs)
Systematic review: (SR)
Transcutaneous electrical nerve stimulation: (TENS)

## 1 Introduction

A case report is a brief article about the personal experience of rare or new medical conditions or effectiveness or harm of certain interventions in patients. In the aspect of safety, a case report is important because it can imply potential association between the specific interventions and the adverse events (AEs) that randomized controlled trials (RCTs) often do not cover [1].

RCTs are the most important research design for assessing the efficacy and safety of an intervention, but there are often concerns presented about missing (or lacking) information about harm-related results [2], and furthermore, it is argued that the RCT is not sufficient to confirm the safety of an intervention due to limited time duration, small sample size, and generalizability issues caused by the strict eligibility criteria of participants [3]. Case reports are a useful tool for identifying potential adverse effects of an intervention, because they are narratives that contain the individual experiences of patients and therapists in the real world [1], and therefore can play a role in raising safety signals about an intervention, and this is recognized as a value that case reports have [4].

The pattern of acupuncture AE reports have been different from medical doctors in the healthcare systems and the acupuncturists. Data indicate that acupuncturists reported adverse effects that occurred during the treatment session, while doctors reported adverse effects occurred after treatment. Delayed doctor-contact because of a first visit to an acupuncturist can be serious if the condition is life-threatening, and effective conventional medical therapy is available and delayed [5]. Acupuncture-related AEs should therefore include reports from both medical doctors, patients, and acupuncturists [6].

The value of a case report is that it can suggest potential association between an intervention and an AE which can provide hypothesis for future cohort studies or case-control studies to determine causality. In addition, it can offer case-based learning for preventing future AEs in clinical practice [1]. However, there has been concerns that anecdotal case reports of suspected interventions often did not provide all the necessary information. For causal assessment and for educational purposes, it would be helpful to have more critical information about the patient's clinical data and the intervention reported in the case reports. So some guidance of case reports on AEs would be necessary in this context [7].

Acupuncture-related AEs can be defined as any unexpected events following acupuncture treatments, which has an established causal relationship between the events and acupuncture as is described for adverse drug reactions [8]. Acupuncture is generally accepted to be a very safe intervention when it is administered in an appropriate clinical setting by well-educated and experienced practitioners [9]. However, potential types of AEs and their relevant risk factors need to be clearly defined and this information is necessary for the development of standard technical guidance for safe acupuncture practice. Case reports are an important source of data for this purpose [10]. Previous studies suggested that essential information for evaluating causality and appropriateness of acupuncture practice was not often reported adequately in the case reports [10,11]. Currently there is no reporting guideline available, hence development of reporting guidelines for accurate and transparent reporting of acupuncture-related AEs case reports is needed. In this systematic review (SR), currently published case reports on acupuncture-related AEs were analyzed to assess whether necessary information is appropriately reported, and which items need to be encouraged to be reported in future. The study objectives of this SR are to assess current publication status and evaluate reporting quality of case reports on the acupuncture-related AEs.

## 2 Methods

This study was a SR of case studies on the acupuncture-related AEs. We located case reports or case series which reported acupuncture-related AEs between 2010 and 2023 (until July) and assessed publication status and appropriateness of reporting of these case studies.

### 2.1 Definition of acupuncture-related adverse events

Definition of acupuncture-related AEs appears differently in the currently available literature [12]. For this study, acupuncture-related AEs were defined based on the International Council for Harmonisation (ICH) definition, “any untoward medical occurrence that may present during treatment with acupuncture but which does not necessarily have a causal relationship with this treatment” [13]. Local reaction in the needling points such as flare and small bleedings as well as aggravations of symptoms, which acupuncture practitioners debated as one of the healing reactions were considered to be acupuncture-related AEs in this review [14]. Serious AEs were defined to be any AEs which introduce life-threating events and intensive treatments are necessary.

### 2.2 Eligibility criteria

The followings were considered for assessing eligibility of the studies.

Population: patients with acupuncture-related AEs

Intervention: Any types of acupuncture regardless of acupuncture needle types, acupuncture points, stimulation methods (manual or electroacupuncture) and style of acupuncture (classical traditional east Asian medicine or western medical acupuncture) were included in this review. Acupuncture used with moxibustion or other types of complementary and alternative medicine (CAM) interventions as well as conventional treatments were also included. Non-needling acupuncture point stimulations including acupressure and transcutaneous electrical nerve stimulation (TENS) were not included in this review. Moxibustion, acupuncture embedding, acupotomy (also known as needle knife therapy, combining the principles of acupuncture and minor surgical procedures), fire needle acupuncture, bee venom acupuncture was excluded because AEs might be introduced through other reasons except acupuncture needling.

Comparator: This item was not considered in this review.

Outcome: We did not consider the clinical outcomes reported in the included case studies.

Study types: Only case reports and case series were included. Some journals publish case reports in the letter to the editors (or correspondence) or an image article for educational purpose and we included this publication as well.

### 2.3 Information sources and searching strategy

Electronic databases including PubMed, Embase, China National Knowledge Infrastructure (CNKI) for Chinese literature and Oriental Medicine Advanced Searching Integrated System (OASIS) for Korean literature were searched and case reports (or case series) published between 2010 and 2023 (July) were included in this review. References included in the published relevant SRs were hand-searched as other resources in addition to the electric database searching. Searching strategy for each database was constructed according to the specific features of each database but important keywords including “acupuncture” for intervention type, “adverse events” and “case report” for study type were properly combined. Detailed searching strategy is suggested in the supplementary file 1.

For the analysis of publication trends, we hand-searched bibliometric information of the included case reports in the Web of Science database only.

### 2.4 Study selection and data-extraction

Two independent authors (T-HK and MSL) accessed eligibility of the located studies independently and any disagreement was discussed until consensus was reached. Summarized information of the included studies were extracted by two authors (T-HK and MSL) including final diagnosis related to AEs based on etiology, patient's information (proceeding conditions or reasons for seeking acupuncture, description about the risk factor for AEs, clinical features of AEs, severity of AEs, clinical outcomes), acupuncture-related information (practitioner type (or certification), needling site, usage of sterile needles, depth of insertion, needle type, stimulation method, acupuncture settings, disinfection procedure) and AE information (time points of acupuncture and AEs, laboratory and pathological findings and other possible causes of AEs) [11]. Bibliometric information including year of publication, authors, country, institution, journals, citation from the included case studies (or series) was extracted.

### 2.5 Analysis

For assessing publication trend of acupuncture-related AE case report, we conducted a bibliometric analysis. Narrative analysis on the information of the included case studies such as most relevant published journals and citations were descriptively analyzed. Bibliometrix package for R (version 4.1.3) were used for this bibliometric analysis. Type of AEs were classified according to the final diagnosis reported in the case reports. Any events related to the systemic or local infection were classified as “Infection”. Any events related to the direct injury of any organs or tissue were classified as “Internal organ or tissue injury” and its subtype was summarized based on the frequently observed organ injuries. “Broken (or retained needles)” were classified when an acupuncture needle was found in the body through examination (or during the surgery) but did not cause any severe damage. “Adverse reactions” were defined as non-specific events after acupuncture treatment such as fainting or hyperventilation [15]. “Other complications” were defined as the events likely to be related to acupuncture and could not be classified elsewhere [10].

The appropriateness of acupuncture practice for the individual case reports was assessed reflecting the conventional acupuncture practice. Acupuncture practice was appraised to be “Appropriate” when all the acupuncture procedures were conducted appropriately considering conventional acupuncture practice; “Inappropriate” when there are potential risk factor related to the acupuncture practice such as practitioners, setting, acupuncture needles etc. So any of acupuncture procedures might be the possible cause of the adverse events or complications considering conventional acupuncture practice. Additionally, when there could still be a definite problem with the acupuncture procedure such as founded broken needles in the body although other acupuncture treatment-related information was insufficient; “Unclear” when there is not enough information for deciding the appropriateness of acupuncture practice.

For assessing whether necessary items of case reports were described in the included studies, the CAse REport guidelines (CARE) checklist was assessed for each study and whether each item was properly described was analyzed and presented narratively [1]. Items in CARE checklist and information of the included case studies (or series) were assessed and graded with “Yes” if all the necessary information was presented appropriately and sufficiently, “No” if there was no available (or insufficient) information in the case study (or series), and not applicable (NA) if the item could not be applicable to the case. Causality between acupuncture and reported AEs in the included studies were evaluated by one Korean medicine doctor with over 10 years of clinical and research experiences. Causality was assessed with WHO-UMC (World Health Organization-Uppsala Monitoring Center) criteria and AE category was modified to five, “Certain”, “Probable”, “Possible”, “Unlikely” and “Unassessable” [16,17]. Only case reports (or series) were included in this review, so risk of bias was not assessed here.

## 3 Results

**Fig. 1:**
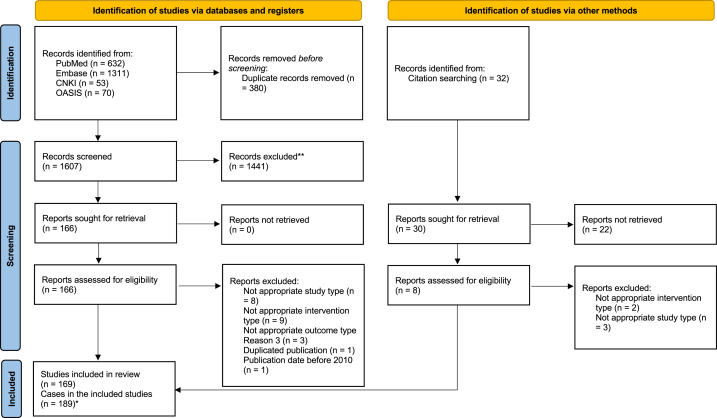
Study flow chart * Numbers of cases included in the studies were counted.

From the electronic database searching and hand-searching, a total of 1607 publication was located initially. Through screening procedure and reviewing of hard copies of eligible publication, 169 case reports (or series) were included in this review [[18], [19], [20], [21], [22], [23], [24], [25], [26], [27], [28], [29], [30], [31], [32], [33], [34], [35], [36], [37], [38], [39], [40], [41], [42], [43], [44], [45], [46], [47], [48], [49], [50], [51], [52], [53], [54], [55], [56], [57], [58], [59], [60], [61], [62], [63], [64], [65], [66], [67], [68], [69], [70], [71], [72], [73], [74], [75], [76], [77], [78], [79], [80], [81], [82], [83], [84], [85], [86], [87], [88], [89], [90], [91], [92], [93], [94], [95], [96], [97], [98], [99], [100], [101], [102], [103], [104], [105], [106], [107], [108], [109], [110], [111], [112], [113], [114], [115], [116], [117], [118], [119], [120], [121], [122], [123], [124], [125], [126], [127], [128], [129], [130], [131], [132], [133], [134], [135], [136], [137], [138], [139], [140], [141], [142], [143], [144], [145], [146], [147], [148], [149], [150], [151], [152], [153], [154], [155], [156], [157], [158], [159], [160], [161], [162], [163], [164], [165], [166], [167], [168], [169], [170], [171], [172], [173], [174], [175], [176], [177], [178], [179], [180], [181], [182], [183], [184], [185], [186]]. The list of excluded studies can be seen in the supplementary file 2. Most studies included single case report, but eleven studies included more than two cases in one publication [23,27,59,79,110,152,154,159,172,180,183] so a total of 189 cases of acupuncture-related AEs were included in the analysis (Fig. 1).

### 3.1 Analysis on the included case reports and publication trends

**Fig. 2:**
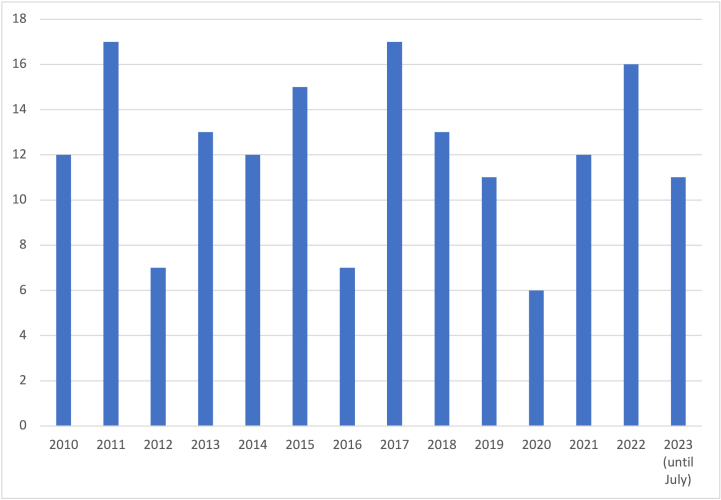
Annual publication numbers of case reports on acupuncture related adverse events.

Between 2010 and 2023 (July), an average of 12 case reports of acupuncture-related AEs were published every year (standard deviation: 3.56) and there was no significant change in the number of papers published each year (Fig. 2). Among the included case reports, the top five countries of the first authors were Korea (n = 42), China (n = 35), US (n = 19), Japan (n = 14) and Taiwan (n = 9, Supplementary Table 1). Regarding article types, publication included case reports (n = 117), letters (n = 26), image articles (n = 17) and others (n = 9, Table 2). Regarding the type of acupuncture-related AEs, internal organ or tissue injury was most frequent (n = 68) followed by infection (n = 60), broken (or retained) needles (n = 23), other complications (n = 14) and adverse reactions (n = 4, Supplementary Table 2). In addition to this, pneumothorax (or hemothorax) was the most frequent patient's diagnosis (n = 27) and other organ or tissue injury (n = 12) and central nervous system injury (n = 11) were also frequent events in the included case reports (Supplementary Table 2). When analyzing authors of the case reports considering the departments and institutions of the authors' affiliations, only 5.6% of publication (n = 8) included at least one authors who might have expertise in acupuncture but medical doctors were only authors of most of the included case reports (n = 131). In addition, there was no case reports where patients were included as authors (Supplementary Table 3). Eighty-one cases did not suggest pre-existing conditions of the reported patients, but others suggested. Fifty cases did not declare any reasons for seeking acupuncture. About the patient's inherent risk factors of AEs, it was well described in 35 cases and partially in 9 cases whereas most of the included cases did not made statement of any information (n = 145). Regarding the clinical outcome of AEs, most cases showed complete recovery with or without sequelae (n = 129). Death cases were rare and bilateral pneumothorax [58,170], pericardial abscess [38], cardiac tamponade acute peritonitis [73], pericarditis [169], cerebral leukemic hemorrhage [96] pulmonary fat embolism [167] and vagus nerve stimulation [160] were reasons for the death (n = 9, Table 1).

**Table 1:** Summary of the included case studies.

ID (first author, year)	Type of AEs*	Final diagnosis related to AEs based on the etiology	Patient's information			
			Proceeding conditions	Reasons for seeking acupuncture	Description about the risk factor for AEs	Clinical outcome (follow up)**
Abe 2022 [18]	Broken (or retained) needles	Broken needle migrated to the intracranial region	No significant medical history	Headache and neck stiffness	NR	R
AlTamimi 2023 [19]	Broken (or retained) needles	Broken needle in the wrist	Hypertension, Diabetes, Hyperthyroidism	NR	NR	R
Bae 2022 [20]	Infection	Coinfection of Sphingomonas paucimobilis meningitis and Listeria monocytogenes bacteremia	Breast cancer	Neck pain	NR	U
Buckley 2011 [21]	Infection	Bacterial endocarditis	Severe atopic eczema	NR	PA	O
Castro-Silva 2011 [22]	Infection	Cutaneous Mycobacterium haemophilum infection	End-stage renal disease secondary to diabetic nephropathy received a kidney transplant	Achilles tendon rupture	W	S
Chiu 2023 [23]	Pneumothorax	Pneumothorax	No systemic diseases	Shoulder and neck pain	W	R
			NR	Shoulder pain	W	R
Cho 2010 [24]	Infection	Mycobacterium abscessus skin infection	NR	NR	NR	R
Cho 2015 [25]	Infection	Gemella morbillorum infection	a thoracic wall contusion because of a bicycle accident	back pain	W	R
Choi 2013 [26]	Infection	Pyogenic liver abscess	Hypertension	Insomnia	NR	R
Chu 2022 [27]	Broken (or retained) needles	Metallic foreign body retained in the body	NR	Neck pain	NR	O
			NR	Neck pain	NR	O
			NR	Neck pain and arm pain	NR	O
Fang 2010 [28]	Adverse reactions	Hyperventilation syndrome	NR	Neck pain	NR	R
Fang 2019 [29]	Ocular injury	Broken needle perforation on eyeball	NR	NR	NR	R
Fielden 2011 [30]	Ocular injury	Ocular perforation	NR	Glaucoma	NR	S
Fleming 2011 [31]	Other complications	Lichen Planus	NR	Low back pain	W	S
Fukaya 2011 [32]	Central nervous system injury	Broken needle perforation on medullar oblongata	NR	Stiff neck	NR	O
Glas 2016 [33]	Infection	Necrotizing fasciitis	NR	NR	NR	S
Gracia-Cazaña 2017 [34]	Infection	Mycobacterium fortuitum infection	NR	NR	NR	R
Guevara-Patiño 2010 [35]	Infection	Mycobacterium fortuitum infection	NR	NR	NR	S
Hampton 2014 [36]	Pneumothorax	Pneumothorax	Cigarette smoking and birth defect requiring a neck brace	Chronic neck pain	W	O
Han 2017 [37]	Infection	*Mycobacterium chelonae* infection	NR	NR	NR	O
Han 2012 [38]	Infection	*Staphylococcus aureus* pericardial abscess	NR	Knee arthralgia	NR	P
Han 2010 [39]	Other organ or tissue injury	Intramuscular Hematoma	Ischemic stroke, atrial fibrillation, warfarin administration	Leg symptom	W	R
Hanabusa 2022 [40]	Hemothorax	Hemothorax	No medical history	Back pain	NR	R
Harrison 2014 [41]	Central nervous system injury	Hypoglossal nerve injury	Sjogrens syndrome, varicose veins	Chronic migraine	W	R
He 2017a [42]	Broken (or retained) needles	Broken needle migration in the abdomen	Cerebral infarction	NR	NR	R
He 2015 [43]	Infection	Pyogenic spondylodiscitis, vertebral osteomyelitis and bilateral psoas abscesses	Rectal cancer, and radical rectectomy and permanent colostomy	Low back pain with lumber disc herniation	W	R
He 2017b [44]	Central nervous system injury	Cervical spinal epidural and subdural hematoma	Hypertension, coronary artery stenosis	Insomnia	W	R
Her 2013 [45]	Heart injury	Cardia tamponade	cerebral infarction and permanent atrial fibrillation on warfarinization	Facial numbness	NR	R
Higgins 2011 [46]	Infection	Disseminated parapox (orf)	atopic eczema	atopic eczema	PA	R
Hong 2018 [47]	Central nervous system injury	Lateral medullary infarction	NR	NR	NR	U
Hong 2015 [48]	Infection	Rice Body Tenosynovitis without Tuberculosis Infection	Gout	Wrist sprain	W	S
Horibe 2016 [49]	Infection	sepsis due to multiple MSSA subcutaneous abscesses	left partial nephrectomy	Lower back pain	NR	S
Horng 2011 [50]	Central nervous system injury	Postdural puncture headache	Healthy	Lower back pain, myofascial pain	NR	R
Hovgaard 2021 [51]	Pneumothorax	Pneumothorax	Chronic neck pain and lower back pain	NR	NR	U
Hsieh 2011 [52]	Infection	Necrotizing Fasciitis by staphylococcus infection	Aplastic anemia, immunosuppressive treatment	Right calf pain	W	S
Hussain 2021 [53]	Pneumothorax	Tension pneumothorax	chronic obstructive pulmonary disease	Back pain	NR	S
Hwang 2013 [54]	Other organ or tissue injury	Acute Gallstone Pancreatitis	NR	Abdominal pain	NR	R
Hwang 2012 [55]	Other organ or tissue injury	Acute pancreatitis	NR	Abdominal pain	NR	R
Inayama 2011 [56]	Pneumothorax	Chylothorax and pneumothorax	NR	NR	NR	R
Jeong 2011 [57]	Broken (or retained) needles	Retained Acupuncture Needle in Lung Parenchyma	Delivery	Lower back pain and shoulder pain	NR	R
Jian 2018 [58]	Pneumothorax	Bilateral tension pneumothorax	NR	Neck and back discomfort	NR	P
Jiang 2023 [59]	Infection	Spinal epidural abscess	NR	Back pain	NR	U
			NR	Shoulder and back pain	NR	U
Jin 2016 [60]	Broken (or retained) needles	Migrated broken needle	NR	Cosmetic acupuncture	NR	R
Jung 2014 [61]	Infection	Cutaneous Mycobacterium massiliense Infection	NR	Chronic low back pain	NR	S
Kang 2018 [62]	Other complications	Pulmonary thromboembolism	NR	Leg pain	NR	O
Kang 2021 [63]	Infection	Recurrent Cellulitis	NR	Lumbar herniated nucleus pulposus	NR	S
Kang 2014 [64]	Broken (or retained) needles	Broken needle in the abdomen	Hypertension and hypothyroidism	NR	NR	R
Kang 2012 [65]	Infection	Psoas abscess and foot ulcer caused by Streptococcus pneumoniae	Diabetes and irregular insulin therapy	Ankle pain	W	S
Kao 2017 [66]	Ocular injury	Penetrating eye injury	Open angle glaucoma	NR	NR	S
Karavis 2015 [67]	Hemothrax	Hemothorax	Heavy smoker, underweight	Stress, musculoskeletal pain	W	R
Kawamura 2023 [69]	Broken (or retained) needles	Broken needle migrated into the cervical spine	NR	NR	NR	R
Kazal 2023 [69]	Broken (or retained) needles	Needle migration into the external auditory canal	NR	Complex regional pain	NR	R
Kennedy 2010 [70]	Pneumothorax	Pneumothorax	Smoking history	Musculoskeletal chest pain	PA	S
Kenz 2012 [71]	Other organ or tissue injury	Thigh hematoma	Warfarin for atrial fibrillation	trochanteric bursitis	W	R
Kewish 2017 [72]	Other complications	Shingles	NR	Chronic low back pain	NR	S
Kim 2017 [73]	Infection	Acute peritonitis	Cervical cancer history	NR	NR	P
Kim 2015a [74]	Broken (or retained) needles	Metallic foreign body retained in the body	Hypertension, Chest pain	Chronic low back and shoulder pain	W	R
Kim 2011a [75]	Infection	Multifocal infection of mycobacterium	NR	Knee pain	NR	R
Kim 2020 [76]	Broken (or retained) needles	Broken needle	NR	Back pain	NR	R
Kim 2010a [77]	Infection	Psoas abscess	Hemodialysis due to renal failure	Lower back pain	PA	R
Kim 2011b [78]	Heart injury	Hemopericardium	NR	Myalgia and dyspepsia	NR	R
Kim 2010b [79]	Infection	Primary Inoculation Tuberculosis	NR	NR	NR	R
			NR	NR	NR	S
			NR	NR	NR	R
Kim 2015b [80]	Infection	Abdominal Wall Actinomycosis	Hypertension, Angina	NR	NR	O
Knudsen 2017 [81]	Infection	*Staphylococcus aureus* Bacteremia	Stroke with sequalae, hypertension	Chronic back pain	NR	S
Kotton 2015 [82]	Infection	Vibrio Vulnificus Necrotizing Fasciitis	Obesity, diabetes mellitus, hyperlipidemia, and non- alcoholic liver cirrhosis	NR	PA	S
Kruse 2019 [83]	Infection	Mycobacterium goodii infection	Hypothyroidism and chronic lymphocytic leukemia … underwent total left knee arthroplasty	Lower back pain	NR	R
Kuo 2011 [84]	Infection	Psoas abscess	NR	Lumbago	NR	S
Kuo 2010 [85]	Other organ or tissue injury	Popliteal Arteriovenous Fistula	No chronic medical history	NR	NR	S
Kwon 2017 [86]	Adverse reactions	Fainting	No specific medical history	Participation of a clinical research	W	R
Larsson 2018 [87]	Pneumothorax	Bilateral pneumothorax	No specific medical history	Shoulder pain	W	R
Lazarow 2017 [88]	Broken (or retained) needles	Migration of retained needles	NR	NR	NR	S
Lee 2013 [89]	Other complications	Myositis ossificans	No specific medical history	Neck pain	W	S
Lee 2019 [90]	Infection	Retroperitoneal abscess with pylephlebitis	No known co- morbidities	Chronic low back pain	NR	R
Lee 2017 [91]	Broken (or retained) needles	Migration of retained needles	Non–ST segment elevation myocardial infarction	General Ache	NR	O
Lee 2021 [92]	Infection	Skin infection	Breast feeding	Tenderness of breast	NR	R
Lee 2022 [93]	Hemopneumothorax	Hemopneumothorax	NR	Musculoskeletal pain	NR	R
Lee 2018 [94]	Ocular injury	Traumatic optic neuropathy	Glaucoma	Glaucoma	NR	S
Lewek 2012 [95]	Broken (or retained) needles	Retention of broken needle	NR	Osteoarthritic back pain	NR	O
Li 2012 [96]	Central nervous system injury	Cerebral Leukemic Hemorrhage	Stomalgia and gingival bleeding	Stomalgia and gingival bleeding	NR	P
Li 2023 [97]	Other organ or tissue injury	Hematoma-mediated colon obstruction	NR	NR	NR	R
Liew 2016 [98]	Other organ or tissue injury	Popliteal artery pseudoaneurysm	NR	Leg muscle pain	NR	S
Liew 2020 [99]	Other organ or tissue injury	Flexor pollicis longus rupture	NR	NR	NR	S
Lim 2013 [100]	Infection	*Vibrio cholerae* septicemia	Liver cirrhosis	Fatigue and lower limb weakness	W	S
Liu 2015 [101]	Infection	Sternoclavicular septic arthritis	Diabetes, hepatitis B-related liver cirrhosis	Sore over nuchal area	NR	R
Liu 2019 [102]	Broken (or retained) needles	Retention of broken needle	No specific medical history	NR	NR	R
Llamas 2020 [103]	Heart injury	Cardiac tamponade	hypersplenism and thrombocytemia	NR	NR	R
Lyu 2022 [104]	Other complications	Pyoderma Gangrenosum	NR	Non healing ulcer of the skin	NR	O
Ma 2018 [105]	Infection	Spinal epidural abscess	Diabetes	lower back pain, leg pain	W	R
Maas 2013 [106]	Infection	*Staphylococcus aureus* sepsis	No specific medical history	Chronic fatigue syndrome	NR	U
Macuha 2010 [107]	Infection	Necrotizing Fasciitis	Osteoarthritis and spinal stenosis	NR	PA	U
May 2015 [108]	Peripheral nerve injury	Superficial radial neuropathy	NR	Shoulder pain	NR	U
McClain 2017 [109]	Broken (or retained) needles	Retention of needle	Hypertension, gastroesophageal reflux disease, and an L-2 vertebroplast	NR	NR	U
Miao 2011 [110]	Other complications	Skin pigmentation	NR	Sciatica	NR	S
			NR	Myofascitis	NR	S
			NR	Insomnia	NR	R
			NR	Lumbar canal stenosis.	NR	S
Millwala 2015 [111]	Infection	Postpartum pyogenic sacroiliitis	Pregnancy	Sciatica	W	S
Miyamoto 2010 [112]	Central nervous system injury	Cervical Cord and Medulla Oblongata Injuries due to Acupuncture	NR	Headache and neck stiffness	NR	R
Mo 2022 [113]	Infection	Thoracic vertebral infection by *Escherichia coli*	Diabetes	Lumbar disk herniation	NR	S
Mohammad 2018 [114]	Pneumothorax	Bilateral tension pneumothorax	No specific medical history	Chronic neck and back pain	PA	R
Nakajima 2010 [115]	Infection	Prosthetic knee infection	Knee pain	NR	NR	S
Narasimhalu 2018 [116]	Central nervous system injury	Inadvertent lumbar puncture	No specific medical history	Lower back pain	NR	R
Narayana 2021 [117]	Infection	Prosthetic knee joint infection	Total knee replacement for osteoarthritis	NR	NR	R
Nguyen 2011 [118]	Pneumothorax	Bilateral Pneumothoraces	Amyotrophic lateral sclerosis (ALS) and hypertension	Chronic low back pain	NR	R
Nishie 2021 [119]	Pneumothorax	Bilateral Pneumothorax	No history of respiratory disease or smoking	Stiff shoulder	W	R
Oncel 2013 [120]	Pneumothorax	Bilateral Pneumothorax	Smoking history	Chronic shoulder pain	W	U
Oskarsson 2017 [121]	Pneumothorax	Bilateral Pneumothoraces	Asthma, hypertension, polycystic kidney disease	NR	NR	U
Pan 2021 [122]	Other complications	Systemic contact dermatitis	NR	Recurrent urticaria	PA	R
Papasotiriou 2014 [123]	Other organ or tissue injury	Rhabdomyolysis and Acute Kidney Injury	Diabetes, hypertension and hypothyroidism	Osteoarthritis of the knees and subsequent arthralgias	NR	R
Park 2013 [124]	Central nervous system injury	Spinal subdural hematoma	Diabetes, hypertension, and antiplatelet therapy	Musculoskeletal pain	NR	R
Park 2019 [125]	Infection	Intracranial abscess	A cranial injury 20 years previously	Occasional headache	W	R
Park 2018 [126]	Other complications	Localized argyria	NR	Epilepsy	NR	U
Prasoppokakorn 2022 [127]	Infection	Mycobacterium massiliense and Scedosporium Infections Superimposed by Tetanus	Falling injury, Schizophrenia	Patient's faith in a medium's ability to communicate with spirits	NR	U
Priola 2019 [128]	Infection	Cranial Epidural Abscess	Crohn disease, immunosuppressive treatment	Occipital neuralgia.	NR	R
Ramalingam 2023 [129]	Pneumothorax	Pneumothorax	Anxiety and depression	NR	NR	R
Ryu 2021 [130]	Broken (or retained) needles	Swallowed needle	Tracheostomy and gastrostomy owing to physical disability	NR	NR	R
Sakai 2017 [131]	Broken (or retained) needles	Migration of embedded needle	NR	Lumbago and uterine myoma	NR	O
Sanchez 2017 [132]	Broken (or retained) needles	Needle retention in the heart	Diabetes, hypertension, hyperlipidemia	NR	NR	U
Sánchez-Cárdenas 2020 [133]	Infection	Cutaneous infection	Previously healthy	Backache	NR	O
Scharf 2015 [134]	Pneumothorax	Pneumothorax	Myasthenia gravis, nonspecific neuropathy, and chronic sinusitis.	Painful neuropathy	W	U
Sfeir 2013 [135]	Infection	*Salmonella typhi* sternal wound infection	NR	a furuncle over chest wall	NR	U
Shew 2019 [136]	Infection	Spinal epidural abscess	Diabetes, diabetic nephropathy with single kidney	Flank and hip pain	PA	U
Shin 2018 [137]	Central nervous system injury	Intraventricular hemorrhage	NR	Neck pain	NR	R
Shuang 2016 [138]	Ocular injury	Perforating injury of eyeball	NR	NR	NR	O
Sia 2018 [139]	Pneumothorax	Traumatic pneumothorax	De Quervain's tenosynovitis	Long-standing neck pain and intermittent numbness and tingling	NR	R
Singh 2015 [140]	Infection	Vertebral Osteomyelitis with Multiple Spinal and Paraspinal Abscesses	Not have any other medical problem	Chronic back pain	NR	R
Smith 2021 [141]	Pneumothorax	Traumatic pneumothorax	Diabetes, Sickle cell trait	Neck pain	W	R
Snyder 2019 [142]	Broken (or retained) needles	Retained broken needle	NR	Chronic neck pain	NR	R
Song 2010 [143]	Broken (or retained) needles	Migration of embedded needle	NR	NR	NR	O
Soumer 2015 [144]	Other organ or tissue injury	Popliteal pseudoaneurysm	NR	Lumbosciatica	NR	R
		Arteriovenous fistula	NR	Osteoarthritis of knee	NR	R
Sreedharan 2012 [145]	Peripheral nerve injury	Posterior interosseous nerve palsy	No known medical history of note	Shoulder pain	NR	S
Sung 2016 [146]	Pneumothorax	Pneumothorax	No known medical history of note	Shoulder pain	W	U
Sung 2021 [147]	Infection	Facial candidal abscesses	No known medical history of note	Blunt eyelid trauma	W	R
Tagami 2013 [148]	Pneumothorax	Bilateral tension pneumothorax	No relevant past medical history	Chronic pain in the cer- vical, thoracic and lumbar regions of the spine	W	R
Talafu 2022 [149]	Other organ or tissue injury	Delayed cardiac tamponade	NR	Cyclomastopathy	NR	P
Tan 2014 [150]	Pneumothorax	Haemopneumothorax	NR	Sore neck	NR	R
Tan 2022 [151]	Other complications	Asymptomatic hyperCKemia	Cerebral infarction, pacreatitis, acetabular fracture, hypertension, hyperlipidemia, diabetes	Sequele of stroke	W	O
Th'ng 2022 [152]	Pneumothorax	Pneumothorax	NR	Muscle ache	NR	U
			NR	Benign paroxysmal positional vertigo	NR	U
			Diabetes, hypertension	Shoulder muscle ache	NR	U
Traeger 2017 [153]	Infection	Mediastinal abscess	NR	a non-penetrative traumatic 5 × 5 cm intramuscular pectoral hematoma	NR	U
Tseng 2013 [154]	Infection	Shoulder infection	NR	NR	NR	S
			Diabetes	NR	NR	S
			NR	NR	NR	S
Tseng 2014 [155]	Infection	Infectious sacroiliitis caused by *Staphylococcus aureus*	No major health problems	Low back pain, A laceration wound on his forehead	NR	R
Tucciarone 2019 [156]	Infection	Pyomyositis of prevertebral muscles	NR	Neck stiffness	NR	U
Ullah 2019 [157]	Infection	Purulent pericarditis masquerading uremic pericarditis	Hypertension, Basal cell skin cancer	Knee arthritis	NR	S
Valgardsson [158]	Pneumothorax	Pneumothorax	Pregnancy	Morning sickness, nausea, vomiting	NR	R
Wang 2023 [159]	Ocular injury	Ocular perforation	NR	Ocular muscle paralysis	NR	O
			NR	Oculomotor paralysis and blepharoptosis	NR	O
Watanabe 2015 [160]	Other complications	Autopsy case of vagus nerve stimulation	NR	NR	NR	P
Weagle 2021 [161]	Pneumothorax	Pneumothorax	Healthy	Low back pain	W	R
Wiggler 2016 [162]	Heart injury	Migrated needle, Cardiac perforation	Healthy	chronic musculo-skeletal pain	NR	R
Wu 2013 [163]	Other complications	Psoriasis flare	Psoriasis	Psoriasis	NR	U
Wu 2022 [164]	Ocular injury	Ocular perforation	NR	Ear disease	NR	U
Xiao 2022 [165]	Infection	Paravertebral abscess and blood stream infection by Burkholderia pseudomallei	Hepatitis B, lumbar disk herniation	NR	W	R
Xu 2021 [166]	Ocular injury	Ocular penetration	NR	Dry eye	NR	U
Xu 2023 [167]	Other complications	Pulmonary fat embolism	Obesity, hypertension, rib fracture due to cardio-pulmonary resuscitation	NR	W	P
Xu 2020 [168]	Heart injury	Cardiac tamponade	Past medical history unremarkable.	Gastric ulcer	NR	U
Yamaguchi 2023 [169]	Infection	Pericarditis due to Pneumococcal Bactermia	Oral steroid for retroperitoneal fibrosis, hypertension, hyperlipidemia	Low back pain and thigh pain	W	P
Yamaguchi 2022 [170]	Pneumothorax	Bilateral pneumothoraces	History of nontuberculous mycobacterium infection	Chronic back pain, stiff shoulders	NR	P
Yang 2014 [171]	Infection	Spinal Epidural Abscess	NR	Hypertrophic intervertebral disc of the lumbar spine	NR	R
Yao 2017 [172]	Infection	Disseminated non-tuberculous mycobacterial infection	Healthy	Lumbar disc herniation”	NR	R
			Without history of heart or lung disease	NR	NR	R
			NR	NR	NR	R
			NR	Osteoarthritis	NR	R
Ye 2014 [173]	Adverse reactions	Vertigo	Insomnia	Constipation	NR	R
Yeo 2015 [174]	Infection	Septic knee arthritis with methicillin-sensitive *Staphylococcus aureus*	NR	Knee osteoarthritis	NR	S
Yi 2011 [175]	Other complications	Electrolysis phenomenon caused by electroacupuncture accident	NR	Upper limb disorder	NR	R
Yoo 2022 [176]	Broken (or retained) needles	Retention of needle	Ankylosing spondylitis	NR	NR	U
Yook 2022 [177]	Broken (or retained) needles	Foreign body granuloma	No underlying disease	Cosmetic purpose	NR	S
You 2014 [178]	Ocular injury	Ocular injury	Laser- assisted in situ keratomileusis (LASIK) surgery	Hemifacial spasms	NR	U
Yu 2013 [179]	Infection	Multiple epidural abscesses	NR	NR	NR	U
Zhang 2014a [180]	Ocular injury	Hyphema	Ocular trauma	Ptosis, diplopia	NR	R
		Conjunctival hemorrhage	Optic neuritis	Optic neuritis	NR	R
Zhang 2014b [181]	Infection	Infratemporal fossa abscess	Trigeminal neuralgia	Trigeminal neuralgia	NR	R
Zhang 2023 [177]	Ocular injury	Ocular penetration	NR	Myopia	NR	S
Zhang 2020 [183]	Adverse reactions	Fainting	Knee pain	NR	NR	R
			left hemiplegia caused by a stroke	NR	NR	R
Zhou 2018 [184]	Other organ or tissue injury	Skin Fistulas Relating to Ascending Colonic Carcinoma	Healthy	Lumbago	NR	S
Zhu 2011 [185]	Other complications	Psoriasis	NR	Red papules and plaques covered with white scales.	NR	U
Zhu 2018 [186]	Central nervous system injury	Medulla oblongata hemorrhage	NR	NR	NR	U

*Type of AEs were classified as Infection, Internal organ or tissue injury, Broken needles, Other complications or adverse reactions. Any events related to the systemic or local infection were classified as Infection. Any events related to the direct injury of any organs or tissue were classified as Internal organ or tissue injury and its subtype was summarized based on the frequently observed organ injuries. Broken (or retained needles) were classified when an acupuncture needle was found in the body through examination (or during the surgery) but did not cause any damage. Adverse reactions were defined as non-specific events after acupuncture treatment such as fainting or hyperventilation. Other complications were defined as events likely to be related to acupuncture that could not be classified elsewhere.

**Clinical outcome represents patient's final status in the final assessment. O-ongoing problems, R-resolved without sequalae, S-resolved with sequalae; P-permanent disability or death; U-unclear.

NR: Not reported, W-well reported, PA-partially reported.

**Table 2:** Type of articles.

Type of article	Numbers	Proportion (%)
Case report	117	69.2%
Letters	26	15.5%
Image article	17	10.0%
Others	9	5.3%
Total	169	100%

Type of articles were classified as a case report, a letter (or correspondence), an image article, or others, which were decided according to the journal's description on the article type.

Through the bibliometric analysis of 116 publications where detailed bibliometric data was available in the Web of Science, Acupuncture in Medicine (n = 14) was found to be the journal that published papers most frequently during the period. This was followed by Annals of dermatology (n = 3), BMC complementary medicine and therapies (n = 3), Internal medicine (n = 3), and Yonsei medical journal (n = 3, Supplementary Table 4). Among the included case reports, the most frequently cited article was “Soft tissue infection due to Mycobacterium fortuitum following acupuncture: a case report and review of the literature” (n = 27) [35], “Acute spinal subdural hematoma with hemiplegia after acupuncture: a case report and review of the literature” was the second (n = 19) [124] and both “Cutaneous Mycobacterium haemophilum infection in a kidney transplant recipient after acupuncture treatment” [22] and “Risks and causes of cervical cord and medulla oblongata injuries due to acupuncture” [112] were the third (n = 15, Supplementary Table 5). When analyzing references of the included case reports, “A cumulative review of the range and incidence of significant adverse events associated with acupuncture” was the most frequently cited reference (n = 20) [187] and “Safety of acupuncture: results of a prospective observational study with 229,230 patients and introduction of a medical information and consent form” [188] and “Systematic review of adverse events following acupuncture: the Japanese literature” [189] were the second (n = 10) (Supplementary Table 6).

### 3.2 Reporting status of acupuncture-related information

When evaluating reporting status of acupuncture-related information, which can be used for assessing appropriateness of acupuncture practice, only a few studies reported necessary information for the assessment of the appropriateness of acupuncture practice in the included case reports. A minority (32.9%) of publications included enough or partial information about practitioners' type and practitioners’ medical background (14.3%), and training history of acupuncture (91.5%) were not suggested in most publications. Detailed information regarding acupuncture treatment, needling site (78.3%) and frequency (and/or duration) of acupuncture (67.2%) were suggested well or partially in most of the included publication. Information assessing causality between the acupuncture practice and AEs such as usage of sterile needles, depth of insertion, needling types, stimulation method and settings of acupuncture practice were not reported appropriately in most of the included publications. The appropriateness of acupuncture practice could be evaluated only in less than half of the case reports (38.2%, Table 3)."

**Table 3:** Reporting status of acupuncture-related information.

Items of acupuncture-related information (n = 189)	Number of well reported studies (%)	Number of partially reported studies (%)	Number of studies without relevant information (%)
Practitioners type (or certification)	45 (23.8%)	16 (8.5.%)	128 (67.7%)
Practitioners' medical background	8 (4.2%)	19 (10.1%)	162 (85.7%)
Practitioners' training history of acupuncture	4 (2.1%)	12 (6.3%)	173 (91.5%)
Needling site	24 (12.7%)	124 (65.6%)	41 (21.7%)
Usage of sterile needles	10 (5.3%)	4 (2.1%)	175 (92.6%)
Frequency and duration of acupuncture treatment	43 (22.8%)	84 (44.4%)	62 (32.8%)
Depth of insertion	14 (7.4%)	6 (3.2%)	169 (89.4%)
Needle type	21 (11.1%)	12 (6.3%)	156 (82.5%)
Stimulation method	18 (9.5%)	5 (2.6%)	166 (87.8%)
Settings of acupuncture practice	5 (2.6%)	36 (19.0%)	148 (78.3%)
Disinfection procedure	6 (3.2%)	2 (1.1%)	181 (95.8%)
	**Appropriate**	**Inappropriate**	**Unclear**
Appraisal for the acupuncture practice*	6 (3.2%)	67 (35.0%)	116 (61.4%)

∗Based on the individual case reports, acupuncture practice was appraised to be Appropriate when all the acupuncture procedures were conducted appropriately considering conventional acupuncture practice; Inappropriate when there are potential risk factor related to the acupuncture practice such as practitioners, setting, acupuncture needles etc. So any of acupuncture procedures might be the possible cause of the adverse events or complications considering conventional acupuncture practice; Unclear when there is not enough information for deciding the appropriateness of acupuncture practice.

### 3.3 Reporting status of adverse event related information

The majority (88.9%) were severe cases which needed intensive treatments or resulted in long-term disability. The cause of AEs was not possible to determine in half of all cases (49.2%), although most of the case reports were well or partially documented for the information to determine temporal sequence of association (87.9%) and the laboratory tests (or pathological analysis) on diagnosis (97.4%). Most of the cases did not suggest analysis on the patient's potential risk factors related to the AEs (68.8%) or other plausible causes of AEs which the patient might had (79.4%). The causal relationship between acupuncture and AEs could not be clearly determined in 23.3% of the case reports (Table 4).

**Table 4:** Reporting status of adverse event related information.

ID (first author, year)	Severity of AEs	Cause of AEs*	Description about the risk factor for AEs	Time relation between acupuncture and AEs	Laboratory or pathological findings	Considering other possible causes of AEs	Causality assessment**
Abe 2022 [18]	Severe	By patient***	NR	W	W	NR	Certain
AlTamimi 2023 [19]	Severe	By practitioner	NR	W	W	NR	Certain
Bae 2022 [20]	Severe	Unclear	W	W	W	NR	Conditional
Buckley 2011 [21]	Severe	By practitioner	W	UA	W	NR	Possible
Castro-Silva 2011 [22]	Severe	By practitioner	W	W	W	NR	Probable
Chiu 2023 [23]	Severe	Unclear	W	W	W	W	Certain
	Severe	Unclear	W	W	W	W	Certain
Cho 2010 [24]	Severe	Unclear	NR	PA	W	NR	Possible
Cho 2015 [25]	Severe	Unclear	W	PA	W	PA	Possible
Choi 2013 [26]	Severe	By practitioner	NR	PA	W	NR	Probable
Chu 2022 [27]	Severe	Unclear	W	UA	W	NR	Possible
	Severe	Unclear	W	UA	W	NR	Possible
	Severe	Unclear	W	UA	W	NR	Possible
	Severe	Unclear	W	UA	W	NR	Possible
Fang 2010 [28]	Mild	Unclear	NR	W	NR	NR	Probable
Fang 2019 [29]	Severe	By practitioner	NR	UA	W	NR	Certain
Fielden 2011 [30]	Severe	By practitioner	NR	UA	W	NR	Possible
Fleming 2011 [31]	Severe	Unclear	W	W	W	W	Probable
Fukaya 2011 [32]	Severe	By practitioner	NR	W	W	NR	Certain
Glas 2016 [33]	Severe	Unclear	W	W	W	NR	Possible
Gracia-Cazaña 2017 [34]	Severe	Unclear	NR	PA	W	NR	Conditional
Guevara-Patiño 2010 [35]	Severe	Unclear	NR	PA	W	NR	Conditional
Hampton 2014 [36]	Severe	By patient	W	W	W	W	Possible
Han 2017 [37]	Severe	Unclear	NR	PA	W	NR	Conditional
Han 2012 [38]	Severe	Unclear	NR	PA	W	NR	Conditional
Han 2010 [39]	Severe	By patient	W	PA	W	W	Probable
Hanabusa 2022 [40]	Severe	Unclear	NR	W	W	W	Certain
Harrison2014 [41]	Severe	Unclear	W	PA	W	NR	Conditional
He 2017a [42]	Severe	By practitioner	NR	W	W	NR	Certain
He 2015 [43]	Severe	By patient	W	W	W	W	Probable
He 2017b [44]	Severe	Unclear	W	W	W	W	Possible
Her 2013 [45]	Severe	By patient	NR	W	W	NR	Certain
Higgins 2011 [46]	Severe	Unclear	W	PA	W	NR	Conditional
Hong 2018 [47]	Severe	Unclear	NR	UA	W	NR	Unlikely
Hong 2015 [48]	Severe	Unclear	W	UA	W	W	Unlikely
Horibe 2016 [49]	Severe	By practitioner	NR	W	W	NR	Possible
Horng 2011 [50]	Severe	By practitioner	NR	W	NR	NR	Unlikely
Hovgaard 2021 [51]	Severe	By patient	NR	UA	W	NR	Probable
Hsieh 2011 [52]	Severe	By patient	W	W	W	W	Probable
Hussain 2021 [53]	Severe	Unclear	NR	W	W	NR	Possible
Hwang 2013 [54]	Severe	By patient	W	W	W	W	Probable
Hwang 2012 [55]	Severe	By practitioner	NR	W	W	NR	Possible
Inayama 2011 [56]	Severe	By practitioner	NR	W	W	NR	Probable
Jeong 2011 [57]	Severe	By practitioner	NR	W	W	NR	Certain
Jian 2018 [58]	Severe	By practitioner	NR	W	W	NR	Probable
Jiang 2023 [59]	Severe	Unclear	NR	W	W	NR	Conditional
	Severe	Unclear	NR	W	W	NR	Conditional
Jin 2016 [60]	Severe	By practitioner	NR	W	W	NR	Certain
Jung 2014 [61]	Severe	Unclear	NR	UA	W	NR	Conditional
Kang 2018 [62]	Severe	By patient	NR	UA	W	NR	Conditional
Kang 2021 [63]	Severe	By practitioner	NR	UA	W	NR	Certain
Kang 2014 [64]	Severe	By practitioner	NR	W	W	NR	Certain
Kang 2012 [65]	Severe	Unclear	W	W	W	W	Possible
Kao 2017 [66]	Severe	By practitioner	NR	W	W	NR	Certain
Karavis 2015 [67]	Severe	Unclear	W	W	W	W	Certain
Kawamura 2023 [69]	Severe	By patient***	NR	W	W	NR	Certain
Kazal 2023 [69]	Severe	Unclear	NR	W	NR	NR	Certain
Kennedy 2010 [70]	Severe	By practitioner	PA	W	W	PA	Certain
Kenz 2012 [71]	Mild	By patient	W	W	W	NR	Probable
Kewish 2017 [72]	Mild	Unclear	NR	W	NR	NR	Unlikely
Kim 2017 [73]	Severe	By practitioner	NR	W	W	NR	Certain
Kim 2015a [74]	Severe	Unclear	W	UA	W	NR	Possible
Kim 2011a [75]	Severe	By practitioner	W	W	W	PA	Possible
Kim 2020 [76]	Severe	By practitioner	NR	W	W	NR	Possible
Kim 2010a [77]	Severe	By patient	PA	W	W	NR	Possible
Kim 2011b [78]	Severe	Unclear	NR	W	W	NR	Certain
Kim 2010b [79]	Severe	By practitioner	NR	PA	W	NR	Certain
	Severe	By practitioner	NR	PA	W	NR	Certain
	Severe	By practitioner	NR	PA	W	NR	Certain
Kim 2015b [80]	Severe	Unclear	NR	PA	W	PA	Possible
Knudsen 2017 [81]	Severe	Unclear	NR	PA	W	NR	Possible
Kotton 2015 [82]	Severe	Unclear	PA	PA	W	NR	Conditional
Kruse 2019 [83]	Severe	Unclear	NR	PA	W	NR	Conditional
Kuo 2011 [84]	Severe	Unclear	NR	PA	W	NR	Conditional
Kuo 2010 [85]	Severe	Unclear	NR	PA	W	NR	Conditional
Kwon 2017 [86]	Mild	By patient	W	W	W	W	Probable
Larsson 2018 [87]	Severe	By practitioner	W	W	W	W	Certain
Lazarow 2017 [88]	Severe	By practitioner	NR	PA	W	NR	Certain
Lee 2013 [89]	Mild	Unclear	W	PA	W	PA	Unlikely
Lee 2019 [90]	Severe	Unclear	NR	PA	W	PA	Possible
Lee 2017 [91]	Severe	By practitioner	NR	UA	W	NR	Certain
Lee 2021 [92]	Mild	By patient***	NR	W	NR	NR	Probable
Lee 2022 [93]	Severe	Unclear	NR	W	W	NR	Certain
Lee 2018 [94]	Severe	By practitioner	NR	W	W	NR	Certain
Lewek 2012 [95]	Severe	By practitioner	NR	W	W	NR	Certain
Li 2012 [96]	Severe	Unclear	W	W	W	NR	Probable
Li 2023 [97]	Severe	Unclear	NR	W	W	NR	Possible
Liew 2016 [98]	Severe	Unclear	NR	PA	W	NR	Conditional
Liew 2020 [99]	Severe	Unclear	NR	W	W	NR	Unlikely
Lim 2013 [100]	Severe	By patient	W	W	W	NR	Possible
Liu 2015 [101]	Severe	Unclear	NR	W	W	NR	Possible
Liu 2019 [102]	Severe	By practitioner	NR	W	W	NR	Certain
Llamas 2020 [103]	Severe	Unclear	NR	W	W	NR	Certain
Lyu 2022 [104]	Severe	Unclear	NR	W	W	NR	Conditional
Ma 2018 [105]	Severe	Unclear	W	PA	W	NR	Conditional
Maas 2013 [106]	Severe	Unclear	NR	PA	W	NR	Conditional
Macuha 2010 [107]	Severe	Unclear	NR	W	W	NR	Conditional
May 2015 [108]	Severe	Unclear	NR	W	W	NR	Conditional
McClain 2017 [109]	Severe	By practitioner	NR	UA	W	NR	Certain
Miao 2011 [110]	Mild	By patient	NR	UA	PA	NR	Certain
	Mild	By patient	NR	UA	PA	NR	Certain
	Mild	By patient	NR	UA	PA	NR	Certain
	Mild	By patient	NR	UA	PA	NR	Certain
Millwala 2015 [111]	Severe	By patient	W	PA	W	W	Conditional
Miyamoto 2010 [112]	Severe	By patient	NR	W	W	NR	Certain
Mo 2022 [113]	Severe	Unclear	NR	W	W	NR	Conditional
Mohammad 2018 [114]	Severe	Unclear	W	W	W	W	Certain
Nakajima 2010 [115]	Severe	By practitioner	NR	W	W	NR	Probable
Narasimhalu 2018 [116]	Severe	Unclear	NR	W	W	NR	Conditional
Narayana 2021 [117]	Severe	Unclear	NR	W	W	NR	Conditional
Nguyen 2011 [118]	Severe	Unclear	NR	W	W	NR	Certain
Nishie 2021 [119]	Severe	Unclear	W	W	W	W	Certain
Oncel 2013 [120]	Severe	Unclear	W	W	W	W	Certain
Oskarsson 2017 [121]	Severe	Unclear	NR	W	W	W	Certain
Pan 2021 [122]	Mild	Unclear	PA	PA	W	NR	Possible
Papasotiriou 2014 [123]	Severe	Unclear	PA	W	W	NR	Possible
Park 2013 [124]	Severe	Unclear	NR	W	W	NR	Possible
Park 2019 [125]	Severe	Unclear	W	PA	W	PA	Possible
Park 2018 [126]	Mild	By practitioner	NR	PA	W	NR	Certain
Prasoppokakorn 2022 [127]	Severe	By patient***	NR	UA	W	NR	Unlikely
Priola 2019 [128]	Severe	Unclear	PA	PA	W	NR	Possible
Ramalingam 2023 [129]	Severe	Unclear	NR	W	W	NR	Conditional
Ryu 2021 [130]	Severe	Unclear	W	W	W	NR	Certain
Sakai 2017 [131]	Mild	By practitioner	NR	PA	W	NR	Certain
Sanchez 2017 [132]	Severe	By practitioner	NR	PA	W	NR	Certain
Sánchez-Cárdenas 2020 [133]	Severe	By practitioner	NR	W	W	NR	Conditional
Scharf 2015 [134]	Severe	Unclear	W	PA	W	NR	Conditional
Sfeir 2013 [135]	Mild	Unclear	NR	PA	W	NR	Conditional
Shew 2019 [136]	Severe	Unclear	PA	PA	W	NR	Possible
Shin 2018 [137]	Severe	By practitioner	NR	W	W	W	Possible
Shuang 2016 [138]	Severe	By practitioner	NR	W	W	W	Certain
Sia 2018 [139]	Severe	By practitioner	W	W	W	NR	Certain
Singh 2015 [140]	Severe	By practitioner	NR	W	W	NR	Probable
Smith 2021 [141]	Severe	By practitioner	W	W	W	W	Certain
Snyder 2019 [142]	Severe	By practitioner	NR	W	W	NR	Certain
Song 2010 [143]	Severe	By practitioner	NR	PA	W	NR	Certain
Soumer 2015 [144]	Severe	Unclear	NR	PA	W	NR	Conditional
	Severe	Unclear	NR	PA	W	NR	Conditional
Sreedharan 2012 [145]	Severe	Unclear	NR	W	W	NR	Conditional
Sung 2016 [146]	Severe	Unclear	W	W	W	NR	Certain
Sung 2021 [147]	Severe	By patient***	W	W	W	NR	Certain
Tagami 2013 [148]	Severe	By practitioner	W	W	W	W	Certain
Talafu 2022 [149]	Severe	Unclear	NR	W	W	NR	Certain
Tan 2014 [150]	Severe	By practitioner	NR	W	W	NR	Certain
Tan 2022 [152]	Mild	By patient	W	W	W	W	Possible
Th'ng 2022 [152]	Severe	By practitioner	NR	W	W	NR	Certain
	Severe	By practitioner	NR	W	W	NR	Certain
	Severe	By practitioner	NR	W	W	NR	Certain
Traeger 2017 [153]	Severe	Unclear	NR	W	W	NR	Possible
Tseng 2013 [154]	Severe	Unclear	NR	W	W	NR	Possible
	Severe	Unclear	NR	W	W	NR	Possible
	Severe	Unclear	NR	W	W	NR	Possible
Tseng 2014 [155]	Severe	Unclear	PA	W	W	PA	Probable
Tucciarone 2019 [156]	Severe	Unclear	NR	W	W	NR	Probable
Ullah 2019 [157]	Severe	Unclear	W	W	W	W	Probable
Valgardsson [158]	Severe	Unclear	NR	W	W	NR	Probable
Wang 2023 [159]	Severe	By practitioner	NR	W	W	NR	Certain
	Severe	By practitioner	NR	W	W	NR	Certain
Watanabe 2015 [160]	Severe	By practitioner	NR	W	W	W	Certain
Weagle 2021 [161]	Severe	Unclear	NR	W	W	W	Probable
Wiggler 2016 [162]	Severe	By practitioner	NR	W	W	NR	Certain
Wu 2013 [163]	Mild	By patient	NR	W	W	NR	Probable
Wu 2022 [164]	Severe	By practitioner	NR	W	W	NR	Probable
Xiao 2022 [165]	Severe	Unclear	W	W	W	W	Conditional
Xu 2021 [166]	Severe	By practitioner	NR	W	W	NR	Certain
Xu 2023 [167]	Severe	Unclear	W	W	W	W	Conditional
Xu 2020 [168]	Severe	Unclear	NR	W	W	NR	Certain
Yamaguchi 2023 [169]	Severe	Unclear	W	W	W	W	Conditional
Yamaguchi 2022 [170]	Severe	By practitioner	NR	W	W	NR	Certain
Yang 2014 [171]	Severe	Unclear	NR	W	W	NR	Possible
Yao 2017 [172]	Severe	By practitioner	NR	W	W	NR	Certain
	Severe	By practitioner	NR	W	W	NR	Certain
	Severe	By practitioner	NR	W	W	NR	Certain
	Severe	By practitioner	NR	W	W	NR	Certain
Ye 2014 [173]	Mild	By patient	NR	W	W	NR	Possible
Yeo 2015 [174]	Severe	Unclear	NR	PA	W	NR	Probable
Yi 2011 [175]	Mild	Unclear	NR	W	W	NR	Certain
Yoo 2022 [176]	Mild	By practitioner	NR	NR	W	NR	Unlikely
Yook 2022 [177]	Severe	By practitioner	NR	NR	W	NR	Certain
You 2014 [178]	Severe	By practitioner	NR	W	W	NR	Certain
Yu 2013 [179]	Severe	Unclear	NR	W	W	NR	Possible
Zhang 2014a [180]	Severe	By practitioner	NR	W	W	NR	Certain
	Severe	By practitioner	NR	W	W	NR	Certain
Zhang 2014b [181]	Severe	By practitioner	NR	W	W	NR	Certain
Zhang 2023 [177]	Severe	By practitioner	NR	W	W	NR	Certain
Zhang 2020 [183]	Mild	By patient	PA	W	W	NR	Possible
Zhou 2018 [184]	Severe	By practitioner	NR	PA	W	NR	Possible
Zhu 2011 [185]	Mild	By patient	PA	W	W	NR	Possible
Zhu 2018 [186]	Severe	Unclear	NR	W	W	NR	Unlikely

*This item is about whether the potential cause of adverse events might be more related to the patient factors or by the practitioner's factors (malpractice or negligence during the acupuncture treatment). If the patient factor is dominant, “By patient”, if the physician factor is dominant, “By practitioner”, and if the cause is difficult to determine, “Unclear”.

**Causality assessment was conducted based on the reported information using WHO-UMC criteria. “Certain” when plausible time relationship between the event and acupuncture was observed in the literature without possible cause of other treatments or underlying diseases for AEs (or complications), “Probable” when reasonable time relationship was observed and AEs (or complications) are unlikely to be explained by other causes, “Possible” when reasonable time relationship was observed and AEs (or complications) were possibly explained by other causes, “Unlikely” when improbable time relationship was observed with other plausible causes, “Conditional” when more data from current undergoing examination was necessary for the evaluation, and “Unassessable” when information was insufficient for judgment.

***Self acupuncture case.

NR: not reported; PA: partially reported; W: well reported; UA: unassessable.

### 3.4 Reporting status of the CARE checklist items

**Fig. 3:**
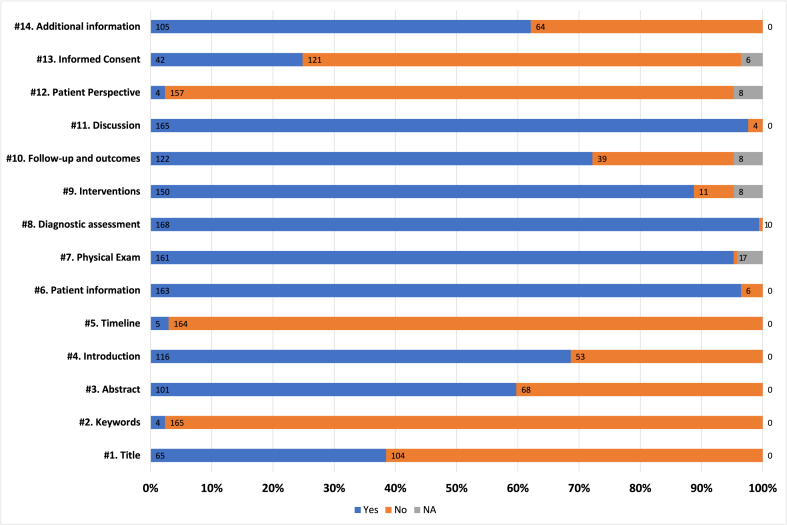
Assessment of the CARE checklist items From #1 to #13, items in the CARE checklist are evaluated. *Additional information is not included in the CARE checklist (2013) and institutional review board (IRB) approval or funding information were assessed in this item. Each item can have “Yes” when all the necessary points are appropriately described or only partially reported when assessed according to the criteria of the CARE statement., “No” when there is no description of the necessary points. “NA” is for the items if it is determined that the information cannot be obtained under normal circumstances. For example, “Patient perspective” and “Informed consent” are not available for autopsy cases.

When assessing complete reporting about acupuncture-related AEs in the included case reports, it was found that a significant number of items were not reported based on the CARE guideline for case reports [190]. “Keywords” (n = 165) and “Timeline” (n = 164), “Patient perspectives” (n = 157) and “Informed consent” (n = 121) were not suggested in most of the case reports. “Patient information” (n = 163), “Clinical finding” (n = 161), “Diagnostic assessment” (n = 168), “Therapeutic interventions” (n = 150), “Follow-up and outcomes” (n = 122) and “Discussion” (n = 165) were comparatively well (or at least partially) reported items of CARE checklist. “Title” (n = 104), “Abstracts” (n = 68) and “Introduction” (n = 53) were items which more than half of the included studies did not present or presented at least in an inappropriate format. In addition to CARE items, about half of the studies provided appropriate additional information such as institutional review board (IRB) approval or funding (n = 105, Fig. 3).

## 4 Discussion

From the included 169 case reports (189 cases), we found that an average 12 case reports on acupuncture-related AEs were published annually. Internal organ or tissue injury (such as pneumothorax, central nervous system injury etc.), and infection were most frequently reported. Most case reports did not include any authors affiliated with an institution where acupuncture practice took place, and the authors were consisted of only medical professionals who have observed and reported the relevant event. Acupuncture-related information such as practitioners' type and training history of acupuncture, usage of sterile needles, depth of insertion, needling types, stimulation method, settings of acupuncture were insufficient. Patient's potential risk factors and other plausible causes were not suggested in most of the studies whereas the causality could not be evaluated in some publications. This might reflect the current situation that there is a lack of expertise to evaluate the appropriateness of acupuncture treatment in the case reports of acupuncture-related AEs. CARE checklist items like “Timeline”, “Keywords”, “Patient perspectives” and “Informed consent” were not appropriately reported in most of the publications.

### 4.1 Comparison with previous studies

Previous reviews on the case reports suggested infections and internal organ or tissue injury including pneumothorax and central nerve system injury as the most frequent conditions of the acupuncture related AEs in the case reports [10,191] This is in line with our result suggesting that internal organ or tissue injury are still most common acupuncture-related AEs in the case reports. The high number of reported pneumothorax indicate a need for continuous attention in clinical practice. Ocular injury emerged as a common acupuncture AE. In addition, broken (or retained) needles were frequently reported, but did not introduce severe organ injury.

About the reporting quality, it was not available to compare the appropriateness of reporting in the previous reviews, because the evaluation of the causality between acupuncture treatment and AEs and whether or not information to determine the appropriateness of acupuncture treatment were absent in the previous review [10]. However, lack of sufficient information in the case report has also been pointed out in previous reviews. In a paper that reviewed case reports of acupuncture-related AEs from 2000 to 2011, it was not possible to identify exactly who performed the acupuncture in most of the case reports included in previous review, nor was it possible to know what qualifications the practitioner had. It was also stated that it was not possible to verify whether appropriate infection prevention measures were taken before the procedure [10]. We also found that the same situation still exists even after 10 years.

Acupuncture-related AEs follow a similar trend compared to the previous 10 years with infections and pneumothorax reported more frequently [10]. In a review that included literature from the previous period of 1981–1994, a similar trend was observed [191]. These might reflect a current need to improve education and practice status of practitioners to ensure safe acupuncture [191]. On the other hand, existing case reports on acupuncture-related AEs did not include enough information to achieve educational purposes [7].

### 4.2 The need for development of a reporting guideline for the case reports of acupuncture-related adverse events

If an AE occurred after acupuncture treatment, did the event and acupuncture have causal relationship truly? Did the patient have any risk factors for the event already? Was the acupuncture practice appropriate for the patient and if not, what action should have been taken for the prevention of the event? What were avoidable components and were not in the acupuncture practice? Detailed information regarding patient condition, AEs and acupuncture practice is necessary for good case reports on the acupuncture-related AEs in terms of educational purpose. In addition to this, key items of the CARE checklist need to be encouraged to be suggested in the acupuncture-related AEs. From this review, we found that any patients who experienced AEs did not participated as authors of the case reports and patient perspectives and informed consent were not reported in most studies, either. Patients' involvement would be important for the case reports on acupuncture-related AEs. Considering these, how can we improve the quality of case reports of AEs of acupuncture? As promoting the usage of the CARE checklist, the necessary items for the acupuncture-related AEs need to be defined, and based on that, a reporting guideline needs to be developed.

### 4.3 Strengths and limitations

This study is an updated review of acupuncture-related AE case reports and shows trend changes of publication compared with the previous review, which is the first strong point. Second, we assessed the appropriateness of reporting, which the previous reviews did not deal with. Third, we suggested authors’ regional distribution, citation information of the published case reports and most frequent journal of the of included case reports through bibliometric analysis, which can present a cross-sectional image of the knowledge ecosystem in this research field. As a result, information of this study can be used for the development of a reporting guideline for case reports of acupuncture-related AEs.

A limitation is that the bibliometric analysis was used for 116 publications because Web of Science did not provide bibliometric information of the other case reports. If these publications were included in the analysis, there could be changes in the bibliometric analysis. Second, there could be another source of publication of acupuncture-related AEs. We could not find any publications where patients involved as the authors of the case reports. This might be introduced from the information source. Only medical journals indexed in major biomedical databases such as Pubmed and Embase were included in the searching strategy. AEs are patient's experiences, so patient's involvement is important. Various types of patient reports, feedback forms, patient advocacy group reports or social media can provide information produced by patients. Next was potential publication bias. From the study findings, Korea and China were the most productive countries for publication of case reports of acupuncture-related AEs and this might be introduced by the limited searching strategy. Previous review included case reports from the other countries in earlier years [191]. Local databases should have been added for preventing potential publication bias. In addition to this, we analyzed the authors' affiliations and department information suggested in the included case reports to assess their expertise and determined whether authors with expertise in acupuncture practice were included. However, we acknowledge that affiliation information may not fully reflect the expertise of all authors, and thus, there may be errors in our analysis. Finally, each journal has different official format for case reports. In this review, about 25% of the included publications were letters or image articles which could not have enough information due to the limited word counts. Some under-reported items might be related to this point.

## 5 Conclusion

In conclusion, information for assessing causality and appropriateness of acupuncture practice was not appropriately suggested in the currently published acupuncture-related AEs. Providing sufficient and appropriate information needs to be promoted in the future case reports, and developing a reporting guideline can be one solution.

## Author contribution statement

Tae-Hun Kim: Conceived and designed the experiments; Performed the experiments; Analyzed and interpreted the data; Wrote the paper. Myeong Soo Lee: Conceived and designed the experiments; Performed the experiments; Analyzed and interpreted the data. Stephen Birch: Conceived and designed the experiments; Performed the experiments; Analyzed and interpreted the data. Terje Alræk: Conceived and designed the experiments; Performed the experiments; Analyzed and interpreted the data. Arne Johan Norheim: Analyzed and interpreted the data. Jung Won Kang: Conceived and designed the experiments; Performed the experiments; Analyzed and interpreted the data.

## Data availability statement

Data will be made available on request.

## Declaration of competing interest

The authors declare the following financial interests/personal relationships which may be considered as potential competing interests:Myeong Soo Lee reports financial support was provided by 10.13039/501100003718KIOM.
