# Supplementary material for: Publication status and reporting quality of case reports on acupuncture-related adverse events: A systematic reviews of case studies
Source: Heliyon. 2023 Sep 30;9(10):e20577. doi: 10.1016/j.heliyon.2023.e20577 (PMC10569964; doi:10.1016/j.heliyon.2023.e20577)
Supplement: Multimedia component 1 [file mmc1.docx]

**Supplementary file 1. Detailed searching strategy**

1. Pubmed

(("Acupuncture"[MeSH Terms] OR "Acupuncture Therapy"[MeSH Terms] OR "acupuncture, ear"[MeSH Terms] OR "Acupuncture Points"[MeSH Terms] OR "Acupuncture Analgesia"[MeSH Terms] OR "Acupuncture"[Title/Abstract]) AND ("adverse effects"[MeSH Subheading] OR ("Injection Site Reaction"[MeSH Terms] OR "Safety Management"[MeSH Terms]) OR ("Patient Harm"[MeSH Terms] OR "Medical Errors"[MeSH Terms]) OR ("bleeding"[Title/Abstract] OR "pain"[Title/Abstract] OR "wheal"[Title/Abstract] OR "swelling"[Title/Abstract] OR "redness"[Title/Abstract] OR "flare"[Title/Abstract] OR "erythema"[Title/Abstract] OR "infection"[Title/Abstract] OR "inflammation"[Title/Abstract] OR "itch"[Title/Abstract] OR "dizziness"[Title/Abstract] OR "paraesthesia"[Title/Abstract] OR "tingling"[Title/Abstract] OR "numbness"[Title/Abstract] OR "aggravation"[Title/Abstract] OR "worsening"[Title/Abstract] OR "sweating"[Title/Abstract] OR "faint"[Title/Abstract] OR "dizzy"[Title/Abstract] OR "cramp"[Title/Abstract] OR "injury"[Title/Abstract] OR "discomfort"[Title/Abstract] OR "malpractice"[Title/Abstract] OR "broken"[Title/Abstract] OR "stuck"[Title/Abstract] OR "forgotten"[Title/Abstract] OR "lost"[Title/Abstract])) AND ("Case Reports"[Publication Type] OR "Single-Case Studies as Topic"[MeSH Terms])) AND (2010:2022[pdat])

1. Embase

#1.  'adverse event'/

#2.  'safety'/exp

#3.  'patient harm'/exp

#4.  'medical error'/exp

#5.  'bleeding'/exp OR bleeding OR pain:ab,ti OR wheal:ab,ti OR swelling:ab,ti OR redness:ab,ti OR flare:ab,ti OR erythema:ab,ti OR infection:ab,ti OR inflammation:ab,ti OR itch:ab,ti OR dizziness:ab,ti OR paraesthesia:ab,ti OR tingling:ab,ti OR numbness:ab,ti OR aggravation:ab,ti OR worsening:ab,ti OR cramp:ab,ti OR injury:ab,ti OR discomfort:ab,ti OR malpractice:ab,ti OR broken:ab,ti OR forgotten:ab,ti OR stuck:ab,ti OR lost:ab,ti

#6.  'acupuncture'/

#7.  'acupuncture device'

#8.  'case study'/exp

#9.  'case report'/exp

#10.  (#1 OR #2 OR #3 OR #4 OR #5) AND (#6 OR #7) AND (#8 OR #9) AND [2010-2022]/py

3. China National Knowledge Infrastrucutre (CNKI)

#1 ( ( 主题%=xls('adverse event') or 题名%=xls('adverse event') or title='adverse event' or v_subject='adverse event' ) AND ( 主题%=xls('acupuncture') or 题名%=xls('acupuncture') or title='acupuncture' or v_subject='acupuncture' ) AND ( 主题%=xls('case') or 题名%=xls('case') or title='case' or v_subject='case' ) )

#2 ( ( 主题%='针刺' or 主题%='針刺' or 题名%='针刺' or 题名%='針刺' ) AND ( 主题%='不良事件' or 题名%='不良事件' ) AND ( 主题%='病例报告' or 主题%='病例報告' or 题名%='病例报告' or 题名%='病例報告' ) )

#3 ( 主题%='针灸' or 主题%='針灸' or 题名%='针灸' or 题名%='針灸' or title=xls('针灸') or title=xls('針灸') or v_subject=xls('针灸') or v_subject=xls('針灸') ) AND ( 主题%='不良事件' or 题名%='不良事件' or title=xls('不良事件') or v_subject=xls('不良事件') ) ) AND ( ( 主要主题='adverse events' ) OR ( 主要主题='a case' ) OR ( 主要主题='adverse event' ) OR ( 主要主题='case report' ) OR ( 主要主题='case series' ) OR ( 主要主题='case reports' ) OR ( 主要主题='tension pneumothorax' ) OR ( 主要主题='bleeding' ) OR ( 主要主题='clinicopathologic' ) OR ( 主要主题='traumatic pneumothorax' ) OR ( 主要主题='consultation' ) OR ( 主要主题='pyoderma gangrenosum' ) )

기간: 2010-2022

1. OASIS

#1 부작용

#2 기흉

#3 이상반응

1. List of publications for the Hand searching

1) T.-H. Kim, J.W. Kang, and W.-S. Park. "The reporting quality of acupuncture-related infections in Korean literature: a systematic review of case studies," Evidence-Based Complementary and Alternative Medicine, vol. 20152015.

2) C.E. Clarkson, D. O'Mahony, and D.E. Jones. "Adverse event reporting in studies of penetrating acupuncture during pregnancy: a systematic review," Acta Obstet Gynecol Scand, vol. 94, no. 5, pp. 453-464, 2015.

3) J.H. Park, J.H. Lee, S. Lee, J.Y. Shin, and T.H. Kim. "Adverse events related to electroacupuncture: a systematic review of single case studies and case series," Acupunct Med, vol. 38, no. 6, pp. 407-416, 2020.

4) S. Xu, L. Wang, E. Cooper, et al. "Adverse events of acupuncture: a systematic review of case reports," Evid Based Complement Alternat Med, vol. 2013, p. 581203, 2013.

**Supplementary file 2. Excluded studies**

| **Study ID** | **Reason** |
| --- | --- |
| Choi 2014 [1] | Not appropriate intervention type (single acupuncture) |
| Ernst 2010 [2] | Not appropriate study type |
| Gao 2019 [3] | Not appropriate outcome type (adverse event) |
| Grusche 2018 [4] | Not appropriate study type |
| Inoue 2011 [5] | Not appropriate intervention type (single acupuncture) |
| Jin 2011 [6] | Not appropriate intervention type (single acupuncture) |
| Kim 2012 [7] | Not appropriate study type |
| Kim 2011 [8] | Not appropriate study type |
| Koh 2010 [9] | Not appropriate study type |
| Lee 2012 [10] | Not appropriate study type |
| Lee 2011 [11] | Not appropriate study type |
| Li 2014 [12] | Not appropriate study type |
| Thomas 1992 [13] | Publication date before 2010 |
| McManus 2018 [14] | Not appropriate intervention type (single acupuncture) |
| Milanlioglu 2011 [15] | Not appropriate study type |
| Neely 2010 [16] | Not appropriate intervention type (single acupuncture) |
| Ota 2020 [17] | Not appropriate study type |
| Penugonda 2011 [18] | Not appripriate outcome type (adverse event) |
| Gustavo 2016 [19] | Not appropriate study type |
| Ronconi 2016 [20] | Not appropriate intervention type (single acupuncture) |
| Uzar 2018 [21] | Not appropriate intervention type (single acupuncture) |
| Wu 2013 [22] | Duplicated publication |
| Yang 2016 [23] | Not appripriate outcome type (adverse event) |
| Yao 2016 [24] | Not appropriate intervention type (single acupuncture) |
| Kim 2018 [25] | Not appropriate study type |
| Zheng 2017 [26] | Not appropriate intervention type (single acupuncture) |
| Zhu 2012 [27] | Not appropriate intervention type (single acupuncture) |

**Reference**

1. Choi HJ: **Cervical necrotizing fasciitis resulting in acupuncture and herbal injection for submental lipoplasty**. *Journal of Craniofacial Surgery* 2014, **25**(5):e507-e509.

2. Ernst E: **Acupuncture - a treatment to die for?** *J R Soc Med* 2010, **103**(10):384-385.

3. Gao XY, Xie J, Meng LY: **Two proved cases of treating herpes zoster pain by needling wei**. *World Journal of Acupuncture - Moxibustion* 2019, **29**(1):28-30.

4. Grusche F, Egerton-Warburton D: **Traumatic pneumothorax after acupuncture: A case series**. *EMA - Emergency Medicine Australasia* 2018, **30**:51.

5. Inoue M, Katsumi Y, Itoi M, Hojo T, Nakajima M, Ohashi S, Oi Y, Kitakoji H: **Direct current electrical stimulation of acupuncture needles for peripheral nerve regeneration: an exploratory case series**. *Acupuncture in Medicine* 2011, **29**(2):88-93.

6. Jin T, Zhang HL, Feng JC: **Concealed cervical extradural hematoma related to small needle-scalpel surgery in China**. *European Journal of Neurology* 2011, **18**:616.

7. Kim DH, Kim SC, Youn HC: **Surgical treatment for intra-thoracic migration of acupuncture needles**. *J Korean Med Sci* 2012, **27**(3):281-284.

8. Kim TH, Kim KH, Kang JW, Lee MS: **RE: Hemopericardium following acupuncture?** *Yonsei Medical Journal* 2011, **52**(2):377-378.

9. Koh SJ, Song T, Kang YA, Choi JW, Chang KJ, Chu CS, Jeong JG, Lee JY, Song MK, Sung HY *et al*: **An outbreak of skin and soft tissue infection caused by Mycobacterium abscessus following acupuncture**. *Clin Microbiol Infect* 2010, **16**(7):895-901.

10. Lee J-H, Cho J-H, Jo D-J: **Cervical epidural abscess after cupping and acupuncture**. *Complementary therapies in medicine* 2012, **20**(4):228-231.

11. Lee J-H, Lee H, Jo D-J: **An acute cervical epidural hematoma as a complication of dry needling**. *Spine* 2011, **36**(13):E891-E893.

12. Li LX, Yin L, He J: **[Case of craniocerebral trauma-induced optic nerve injury]**. *Zhongguo Zhen Jiu* 2014, **34**(5):454.

13. List T, Helkimo M, Lapeer GL: **Adverse Events of Acupuncture and Occlusal Splint Therapy in the Treatment of Craniomandibular Disorders**. *CRANIO®* 1992, **10**(4).

14. McManus R, Cleary M: **Radial nerve injury following dry needling**. *BMJ Case Rep* 2018, **2018**.

15. Milanlıoğlu A, Torlak PT: **Intracranial hemorrhage and cerebellar infarction caused by acupuncture**. *Neurology India* 2011, **59**(2).

16. Neely D, Jeganathan R, Campalani G: **Transcaval migration of an acupuncture needle from the abdominal cavity to the heart**. *J Card Surg* 2010, **25**(6):654-656.

17. Ota K, Yokoyama H, Takasu A: **Discovery of decades-old acupuncture needle fragments during routine care for an arm injury**. *Acute Medicine and Surgery* 2020, **7**(1).

18. Penugonda B, Kaplan J, Goddard G: **Chronically retained acupuncture needles**. *Medical Acupuncture* 2011, **23**(2):115-118.

19. Pereira G, Mesquita A, Martins-da-Encarnação AP: **Adverse events during acupuncture training at the 3rd Edition of the Post-Graduation on Medical Acupuncture at Health Sciences School of the University of Minho**. *Acupuncture and Related Therapies* 2016, **4**(1-3).

20. Ronconi G, De Giorgio F, Ricci E, Maggi L, Spagnolo AG, Ferrara PE: **[Pneumothorax following dry needling treatment: legal and ethical aspects]**. *Ig Sanita Pubbl* 2016, **72**(5):505-512.

21. Uzar T, Turkmen I, Menekse EB, Dirican A, Ekaterina P, Ozkaya S: **A case with iatrogenic pneumothorax due to deep dry needling**. *Radiology Case Reports* 2018, **13**(6):1246-1248.

22. Wu JJ, Caperton C: **Images in clinical medicine. Psoriasis flare from Koebner's phenomenon after acupuncture**. *N Engl J Med* 2013, **368**(17):1635.

23. Yang X: **Case of epilepsy after cerebral hemorrhage caused by traumatism**. *Zhongguo zhen jiu = Chinese acupuncture & moxibustion* 2016, **36**(5):560.

24. Yao Y, Hong W, Chen H, Guan Q, Yu H, Chang X, Yu Y, Xu S, Fan W: **Cervical spinal epidural abscess following acupuncture and wet-cupping therapy: A case report**. *Complement Ther Med* 2016, **24**:108-110.

25. Youn-Jung K, Sung-Han K, Jin LH, Young KW: **Infectious Adverse Events Following Acupuncture: Clinical Progress and Microbiological Etiology**. *Journal of Korean medical science* 2018, **33**(24).

26. Zheng J, Deng M, Qiu X, Chen Z, Li D, Deng X, Deng Q, Yu Z: **Rhabdomyolysis, lactic acidosis, and multiple organ failure during telbivudine treatment for hepatitis B: A case report and review of the literature**. *Journal of Medical Case Reports* 2017, **11**(1).

27. Zhu HG, Zha BS, Liu B: **Aortic intramural hematoma presenting as paraplegia progressed into segmental aortic dissection**. *Thoracic and Cardiovascular Surgeon* 2012, **60**(8):548-551.

**Supplementary table 1. Frequencies of the country of the first authors of the included case reports**

|  | | | | | | |
| --- | --- | --- | --- | --- | --- | --- |
| **Country** | | **Counts** | | **% of Total** | |  |
| Korea |  | 42 |  | 25.0 % |  |  |
| China |  | 35 |  | 20.8 % |  |  |
| US |  | 19 |  | 11.3 % |  |  |
| Japan |  | 14 |  | 8.3 % |  |  |
| Taiwan |  | 10 |  | 6.0 % |  |  |
| UK |  | 6 |  | 3.6 % |  |  |
| Singapore |  | 6 |  | 3.6 % |  |  |
| Denmark |  | 4 |  | 2.4 % |  |  |
| Malaysia |  | 4 |  | 2.4 % |  |  |
| Canada |  | 3 |  | 1.8 % |  |  |
| Spain |  | 3 |  | 1.8 % |  |  |
| Australia |  | 2 |  | 1.2 % |  |  |
| Greece |  | 2 |  | 1.2 % |  |  |
| Netherlands |  | 2 |  | 1.2 % |  |  |
| New Zealand |  | 2 |  | 1.2 % |  |  |
| Afghanistan |  | 1 |  | 0.6 % |  |  |
| Brazil |  | 1 |  | 0.6 % |  |  |
| Iceland |  | 1 |  | 0.6 % |  |  |
| Ireland |  | 1 |  | 0.6 % |  |  |
| Israel |  | 1 |  | 0.6 % |  |  |
| Lebanon |  | 1 |  | 0.6 % |  |  |
| Mexico |  | 1 |  | 0.6 % |  |  |
| Poland |  | 1 |  | 0.6 % |  |  |
| Saudi Arabia |  | 1 |  | 0.6 % |  |  |
| Switzerland |  | 1 |  | 0.6 % |  |  |
| Thailand |  | 1 |  | 0.6 % |  |  |
| Tunis |  | 1 |  | 0.6 % |  |  |
| Turkey |  | 1 |  | 0.6 % |  |  |
| Venezuela |  | 1 |  | 0.6 % |  |  |
| Total |  | 168 |  | 100 % |  |  |
|  | | | | | | |

**Supplementary table 2. Type of adverse events**

| Type of adverse events |  |  | Number of case reports |
| --- | --- | --- | --- |
| Infection |  |  | 60 |
| Internal organ or tissue injury |  |  | 68 |
|  | Pneumothorax (including hemothorax) | 27 |  |
|  | Central nervous system injury | 11 |  |
|  | Other organ or tissue injury | 12 |  |
|  | Ocular injury | 11 |  |
|  | Heart injury | 5 |  |
|  | Peripheral nerve injury | 2 |  |
| Broken (or retained) needles |  |  | 23 |
| Other complications |  |  | 14 |
| Adverse reactions |  |  | 4 |
| Sum |  |  | N = 169 |

**Supplementary table 3. Analysis on the inclusion of acupuncture experts in the author list**

| **Type of reporters** | **Number** | **Proportion** |
| --- | --- | --- |
| A | 14 | 8.3 % |
| P | 0 | 0 % |
| MD | 149 | 88.2 % |
| U | 6 | 3.5 % |
| Total | 169 | 100 % |

A-Included anyone in the authors list who might have expertise in acupuncture considering his (or her) department or institution, P- Included any patients in the authors list, MD-Included only medical practitioners in the authors list, U-unidentified

**Supplementary table 4. Most relevant journals (Top 17)**

| Sources | Articles |
| --- | --- |
| ACUPUNCTURE IN MEDICINE | 14 |
| ANNALS OF DERMATOLOGY | 3 |
| BMC COMPLEMENTARY MEDICINE AND THERAPIES | 3 |
| INTERNAL MEDICINE | 3 |
| YONSEI MEDICAL JOURNAL | 3 |
| ASIAN JOURNAL OF SURGERY | 2 |
| BMC SURGERY | 2 |
| CUREUS | 2 |
| FORENSIC SCIENCE INTERNATIONAL | 2 |
| JOURNAL OF ALTERNATIVE AND COMPLEMENTARY MEDICINE | 2 |
| JOURNAL OF EMERGENCY MEDICINE | 2 |
| KAOHSIUNG JOURNAL OF MEDICAL SCIENCES | 2 |
| KOREAN JOURNAL OF INTERNAL MEDICINE | 2 |
| MEDICINE | 2 |
| OPHTHALMOLOGY | 2 |
| SPINE JOURNAL | 2 |
| WORLD NEUROSURGERY | 2 |

**Supplementary table 5. Most cited articles (Top 20)**

| Title | Cited articles | Total Citations |
| --- | --- | --- |
| Soft tissue infection due to Mycobacterium fortuitum following acupuncture: a case report and review of the literature | GUEVARA-PATINO A, 2010, J INFECT DEV COUNTR | 27 |
| Acute spinal subdural hematoma with hemiplegia after acupuncture: a case report and review of the literature | PARK J, 2013, SPINE J | 19 |
| Cutaneous Mycobacterium haemophilum infection in a kidney transplant recipient after acupuncture treatment | CASTRO-SILVA AN, 2011, TRANSPL INFECT DIS | 15 |
| Risks and causes of cervical cord and medulla oblongata injuries due to acupuncture | MIYAMOTO S, 2010, WORLD NEUROSURG | 15 |
| Three cases of primary inoculation tuberculosis as a result of illegal acupuncture | KIM JK, 2010, ANN DERMATOL | 13 |
| Teaching NeuroImages: multiple epidural abscesses after acupuncture | YU HJ, 2013, NEUROLOGY | 12 |
| Acupuncture-induced haemothorax: a rare iatrogenic complication of acupuncture | KARAVIS MY, 2015, ACUPUNCT MED | 10 |
| Hemopericardium following acupuncture | KIM JH, 2011, YONSEI MED J | 9 |
| Bilateral tension pneumothorax related to acupuncture | TAGAMI R, 2013, ACUPUNCT MED | 9 |
| Staphylococcus aureus endocarditis as a complication of acupuncture for eczema | BUCKLEY DA, 2011, BRIT J DERMATOL | 9 |
| Cutaneous Mycobacterium massiliense infection of the sole of the feet | JUNG MY, 2014, ANN DERMATOL | 8 |
| Acupuncture-Induced Cranial Epidural Abscess: Case Report and Review of the Literature | PRIOLA SM, 2019, WORLD NEUROSURG | 8 |
| Salmonella typhi sternal wound infection | SFEIR M, 2013, AM J INFECT CONTROL | 8 |
| Localized argyria: troublesome side-effect of acupuncture | PARK MY, 2018, J EUR ACAD DERMATOL | 8 |
| Autopsy diagnosis of acupuncture-induced bilateral tension pneumothorax using whole-body postmortem computed tomography: A case report | JIAN JQ, 2018, MEDICINE | 7 |
| Serratia marcescens spinal epidural abscess formation following acupuncture | YANG CW, 2014, INTERNAL MED | 7 |
| Acupuncture‐induced Popliteal Arteriovenous Fistula Successfully Treated With Percutaneous Endovascular Intervention | KUO HF, 2010, KAOHSIUNG J MED SCI | 6 |
| Myositis ossificans in the paraspinal muscles of the neck after acupuncture: a case report | LEE DG, 2013, SPINE J | 6 |
| An acupuncture needle remaining in a lung for 17 years: case study and review | LEWEK P, 2012, ACUPUNCT MED | 6 |
| Needle acupuncture-induced Koebner phenomenon in a psoriatic patient | ZHU LL, 2011, J ALTERN COMPLEM MED | 6 |

**Supplementary table 6. Most cited references (Top 20)**

| **Title** | **Cited References** | **Citations** |
| --- | --- | --- |
| [A cumulative review of the range and incidence of significant adverse events associated with acupuncture](https://journals.sagepub.com/doi/abs/10.1136/aim.22.3.122) | WHITE ADRIAN, 2004, ACUPUNCT MED, V22, P122 | 20 |
| [Safety of acupuncture: results of a prospective observational study with 229,230 patients and introduction of a medical information and consent form](https://www.karger.com/article/Abstract/209315) | WITT CM, 2009, FORSCH KOMPLEMENTMED, V16, P91, DOI 10.1159/000209315 | 10 |
| Systematic review of adverse events following acupuncture: the Japanese literature | YAMASHITA H, 2001, COMPLEMENT THER MED, V9, P98, DOI 10.1054/CTIM.2001.0446 | 10 |
| Is acupuncture safe? A systematic review of case reports | LAO LX, 2003, ALTERN THER HEALTH M, V9, P72 | 9 |
| [Paraplegia caused by spinal infection after acupuncture](https://www.nature.com/articles/3101819) | BANG MS, 2006, SPINAL CORD, V44, P258, DOI 10.1038/SJ.SC.3101819 | 8 |
| [Prospective studies of the safety of acupuncture: a systematic review](https://www.sciencedirect.com/science/article/pii/S0002934301006519) | ERNST E, 2001, AM J MED, V110, P481, DOI 10.1016/S0002-9343(01)00651-9 | 7 |
| [A migrated acupuncture needle in the medulla oblongata](https://pubmed.ncbi.nlm.nih.gov/15477518/) | HAMA Y, 2004, ARCH NEUROL-CHICAGO, V61, P1608, DOI 10.1001/ARCHNEUR.61.10.1608 | 6 |
| Migration of an acupuncture needle into the medulla oblongata | ABUMI K, 1996, EUR SPINE J, V5, P137, DOI 10.1007/BF00298396 | 5 |
| Subarachnoid hemorrhage and spinal root injury caused by acupuncture needle--case report | MURATA K, 1990, NEUROLOGIA MEDICO-CHIRURGICA, V30, P956, DOI 10.2176/NMC.30.956 | 5 |
| [Is pneumothorax after acupuncture so uncommon?](https://pubmed.ncbi.nlm.nih.gov/23991325/) | STENGER M, 2013, J THORAC DIS, V5, PE144, DOI 10.3978/J.ISSN.2072-1439.2013.08.18 | 5 |
| Safety of Acupuncture: Overview of Systematic Reviews | CHAN MWC, 2017, SCI REP-UK, V7, DOI 10.1038/S41598-017-03272-0 | 4 |
| Cardiac tamponade caused by acupuncture: a review of the literature | ERNST E, 2011, INT J CARDIOL, V149, P287, DOI 10.1016/J.IJCARD.2010.10.016 | 4 |
| An official ATS/IDSA statement: diagnosis, treatment, and prevention of nontuberculous mycobacterial diseases | GRIFFITH DE, 2007, AM J RESP CRIT CARE, V175, P367, DOI 10.1164/RCCM.200604-571ST | 4 |
| [Fatal cardiac tamponade after acupuncture through congenital sternal foramen](https://www.thelancet.com/journals/lancet/article/PIIS0140-6736(95)91004-2/fulltext) | HALVORSEN TB, 1995, LANCET, V345, P1175, DOI 10.1016/S0140-6736(95)91004-2 | 4 |
| [Adverse Events Following Acupuncture: A Systematic Review of the Chinese Literature for the Years 1956–2010](https://www.liebertpub.com/doi/full/10.1089/acm.2011.0825) | HE WJ, 2012, J ALTERN COMPLEM MED, V18, P892, DOI 10.1089/ACM.2011.0825 | 4 |
| An autopsy case of bilateral tension pneumothorax after acupuncture | IWADATE KIMIHARU, 2003, LEG MED (TOKYO), V5, P170, DOI 10.1016/S1344-6223(03)00052-X | 4 |
| [The placebo effect in alternative medicine: can the performance of a healing ritual have clinical significance?](https://www.acpjournals.org/doi/abs/10.7326/0003-4819-136-11-200206040-00011) | KAPTCHUK TJ, 2002, ANN INTERN MED, V136, P374, DOI 10.7326/0003-4819-136-5-200203050-00010 | 4 |
| Cardiac tamponade caused by penetration of an acupuncture needle into the right ventricle | KATAOKA H, 1997, J THORAC CARDIOV SUR, V114, P674, DOI 10.1016/S0022-5223(97)70060-6 | 4 |
| [Incidence of iatrogenic pneumothorax following acupuncture treatments in Taiwan](https://journals.sagepub.com/doi/abs/10.1136/acupmed-2018-011697) | LIN SK, 2019, ACUPUNCT MED, V37, P332, DOI 10.1136/ACUPMED-2018-011697 | 4 |
